# Perampanel-induced depressive disorder in an adolescent with temporal lobe epilepsy: balancing seizure control and psychiatric safety

**DOI:** 10.1192/j.eurpsy.2026.11119

**Published:** 2026-07-31

**Authors:** J. Sun, D. V. Tan

**Affiliations:** Department of Developmental Psychiatry, Institute of Mental Health, Singapore, Singapore

## Abstract

**Introduction:**

Perampanel is a selective, non-competitive antagonist of the AMPA receptor, acting to modulate excitatory glutamatergic neurotransmission (Steinhoff, 2015), which contributes to its antiepileptic efficacy. Glutamatergic activity within fronto-limbic networks - particularly the amygdala, orbitofrontal cortex, and prefrontal regions - is essential for emotional regulation, behavioural control, and impulse modulation. Dysregulation in these pathways have been associated with heightened aggression, impulsivity, and mood dysregulation (Coccaro et al., 2013). Adverse effects such as irritability, aggression, and hostility have been reported in roughly 20% of patients prescribed with perampanel (Mammì et al., 2022). We describe a perampanel induced depressive disorder in an adolescent with temporal lobe epilepsy.

**Objectives:**

To illustrate psychiatric side effects of perampanel in adolescent temporal lobe epilepsy and the need for psychiatric monitoring during treatment.

**Methods:**

Clinical and corroborative histories were obtained. Informed consent was obtained and information was de-identified to ensure anonymity. She was followed up for 4 months.

**Results:**

A 17-year-old female with temporal lobe epilepsy and no past psychiatric history, previously managed with valproate and levetiracetam, was switched to perampanel to optimise seizure control. After an increase in perampanel dose 3 months prior to presentation, she experienced marked depressive symptoms. She presented to emergency services for an impulsive suicide attempt and aggression towards family. While there have been ongoing psychosocial stressors such as financial and academic concerns, she had no prior history of aggression or suicidal behaviour. Following discontinuation of perampanel and resumption of valproate, along with sertraline 25mg daily, there was significant improvement in her mood. Sertraline was stopped successfully. Despite ongoing breakthrough seizures once weekly, mood and overall functioning improved remarkably following mood stabilization.

**Conclusions:**

Temporal link between onset of depressive symptoms and perampanel titration, along with clinical improvement following cessation, suggests a perampanel induced depressive disorder. While existing studies have described aggression in adults, reports in adolescents remain uncommon (Ettinger et al., 2015). Predisposing factors such as psychosocial vulnerability, co-administration of levetiracetam may have increased her susceptibility to depressive effects of perampanel. This case highlights the importance of psychiatric monitoring during perampanel titration in adolescents, especially with anti-epileptic polypharmacy. Multidisciplinary collaboration remains critical to monitor neuropsychiatric side effects and optimize patient outcomes.

**Disclosure of Interest:**

None Declared

